# Gas Sensing with Iridium Oxide Nanoparticle Decorated Carbon Nanotubes

**DOI:** 10.3390/s19010113

**Published:** 2018-12-31

**Authors:** Juan Casanova-Cháfer, Eric Navarrete, Xavier Noirfalise, Polona Umek, Carla Bittencourt, Eduard Llobet

**Affiliations:** 1 MINOS-EMaS, Universitat Rovira i Virgili, 43007 Tarragona, Spain; juan.casanova@urv.cat (J.C.-C.); eric.navarrete@urv.cat (E.N.); 2 Materia Nova, 7000 Mons, Belgium; xavier.noirfalise@umons.ac.be; 3 Jožef Stefan Institute, 10000 Ljubljana, Slovenia; polona.umek@ijs.si; 4 ChIPS, University of Mons, 7000 Mons, Belgium; carla.bittencourt@umons.ac.be

**Keywords:** iridium oxide, carbon nanotubes, chemoresistive gas sensor, metal nanoparticles, relative humidity effect

## Abstract

The properties of multi-wall carbon nanotubes decorated with iridium oxide nanoparticles (IrO_x_-MWCNTs) are studied to detect harmful gases such as nitrogen dioxide and ammonia. IrO_x_ nanoparticles were synthetized using a two-step method, based on a hydrolysis and acid condensation growth mechanism. The metal oxide nanoparticles obtained were employed for decorating the sidewalls of carbon nanotubes. Iridium-oxide nanoparticle decorated carbon nanotube material showed higher and more stable responses towards NH_3_ and NO_2_ than bare carbon nanotubes under different experimental conditions, establishing the optimal operating temperatures and estimating the limits of detection and quantification. Furthermore, the nanomaterials employed were studied using different morphological and compositional characterization techniques and a gas sensing mechanism is proposed.

## 1. Introduction

Chemical sensors employing carbon nanomaterials, like carbon nanotubes and graphene, have attracted great research interest. Specifically, carbon nanotubes have been extensively employed in gas sensing applications due to their suitable electronic, physical, and chemical properties, such as nanometer-size, high carrier mobility, and surface area to volume ratio [1]. Additionally, by functionalizing the sidewalls of multi-wall carbon nanotubes (MWCNT), some sensing properties can be enhanced, such as reproducibility, selectivity, and sensitivity [2]. Different options have been widely used to improve gas sensing performance, such as grafting functional groups onto the carbon nanotubes (CNT) surface [3] or decorating them with metal or metal oxide nanoparticles [4]. Nanoparticles present some advantages like high surface area, control over the local environment, and improved mass transport that cannot be achieved at bulk level [5].

Even though many papers have been published on the attachment of metal or metal oxide nanoparticles on carbon nanotube sidewalls [4], to the best of our knowledge this is the first time that the decoration of carbon nanotubes with iridium oxide nanoparticles in chemoresistive gas sensing is studied. For that reason, this paper reports the improvements obtained by loading MWCNTs with IrO_x_ nanoparticles.

Iridium oxide has been reported for electrochemical sensing and catalyst applications, especially for water splitting at low or moderate temperatures [6] due to its ideal properties such as high catalytic activity, stability, and selectivity under specific reaction conditions [5]. In addition, IrO_x_ was employed in biosensing applications [7,8] and as a pH sensor [9,10]. Iridium oxide behaves as a p-type semiconductor with medium band gap energy (3.12 eV) and charge carrier concentration of 4.2 × 10^21^ cm^−3^ [11]. In addition, iridium, has been employed to modify different metal oxides for gas sensing. Even if metal oxides are loaded with iridium metal, it appears in the form of iridium oxide nanoparticles at the high operating temperatures of metal oxide gas sensors. Iridium oxide has been reported to catalyze gallium oxide for detecting ethanol and propane at 600 °C [12], combined to tungsten oxide for detecting ethanol [13], added to titanium oxide films for detecting oxygen [14], or employed in combination with tin oxide in an attempt to diminish moisture cross-sensitivity in the detection of carbon monoxide [15].

As already reported [16], the presence of oxygenated defects and functional groups (e.g., carboxylic acid) on the sidewalls of MWCNTs helps in achieving a stable grafting of metal or metal oxide nanoparticles to carbon nanotubes and enables an efficient charge carrier transfer between them. Therefore, this approach was also employed here for the grafting IrO_x_ nanoparticles onto carbon nanotubes in view of developing simple, low cost chemoresistive gas sensors.

The hybrid nanomaterial and gas sensors were characterized employing different techniques such as Raman spectroscopy, transmission electron microscopy (TEM), scanning electron microscopy (SEM), and X-ray photoelectron spectroscopy (XPS) to elucidate morphology and chemical composition. The resistance changes during the exposure to different contaminant gases under different experimental conditions were measured.

While it is well-known that carbon nanomaterials show remarkable sensitivity to nitrogen dioxide, the performance of metal or metal oxide NP decorated CNTs in the detection of ammonia vapors remains rather poor. Probably this is due to the significantly weaker affinity between NH_3_ and carbon nanotubes and its associated poor charge transfer efficiency [17]. However, the capability of IrO_x_ to detect NH_3_ has been reported [18] and for that reason, in this paper we explore the possibility of employing IrO_x_-MWCNTs as gas-sensitive material for detecting NO_2_ and NH_3_.

## 2. Materials and Methods

### 2.1. Material Synthesis

The synthesis of iridium oxide (IrO_x_) nanoparticles was achieved following the method proposed by Zhao [19], in which a solution of iridium oxide nanoparticles is prepared via a two-step process (Equation (1)). The first step consists of the preparation of 2 mM dissolution using potassium hexachloroiridate (IV) (K_2_IrCl_6_) in 100 mL of distilled water. Then, pH was adjusted to 13 employing sodium hydroxide (NaOH) diluted in distilled water and the product was heated to 90 °C during 30 min to ensure the total hydrolysis of K_2_IrCl_6_, breaking the Ir–Cl bonds and creating the complex [Ir(OH)_6_]^−2^. This resulted in a color change of the dissolution from red-brown (K_2_IrCl_6_) to yellow ([Ir(OH)_6_]^−2^) (see Equation (1)).

An acid condensation process was conducted in the second step, in which a 3 M nitric acid (HNO_3_) dissolution was employed to decrease the pH to 1 under vigorous stirring during 90 min in order to avoid the formation of precipitates. In this step, the ([Ir(OH)_6_]^−2^) complex forms ligand-free IrO_x_ nanoparticles dispersed in water, with their characteristic deep blue color (see Figure S1 Supplementary Materials) due to the protonation and condensation of ([Ir(OH)_6_]^−2^). Once the iridium nanoparticles are formed, it is important to store the dissolution at 2 °C to avoid the formation of precipitates.

(1) $${\left[{\mathrm{IrCl}}_{6}\right]}^{-2}\;\overset{{\mathrm{OH}}^{-}\;}{\to }\;{\left[\mathrm{Ir}{\left(\mathrm{OH}\right)}_{6}\right]}^{-2}\;\overset{H^{+}}{\to }\;{\mathrm{IrO}}_{x}{+\mathrm{nH}}_{2}O$$

Once the metal oxide nanoparticles had been obtained, the surface of MWCNTs was decorated by attaching the IrO_x_ nanoparticles via an impregnation technique. The impregnation technique consists of the dropwise addition of metal nanoparticles to a MWCNTs solution heated at 80 °C under vigorous stirring. Functionalized MWCNTs were purchased from Nanocyl S.A. (Belgium) with carbonyl and carboxyl functional groups grafted to their surface because these MWCNTs had undergone a cold plasma treatment. As already stated, the presence of these functional groups on the carbon nanotubes sidewalls helps attaching the iridium oxide nanoparticles onto their outer wall.

### 2.2. Material Characterization

The nanomaterials obtained were analyzed employing several techniques. For instance, both the crystallinity of carbon nanotubes and the confirmation of the presence of iridium oxide nanoparticles in the hybrid samples were evaluated using Raman Spectroscopy. This analysis was performed using a Raman spectrometer from Renishaw, plc. (Wotton-under-Edge, UK), which was coupled to a confocal Leica DM2500 microscope. The laser employed had a wavelength of 514 nm.

The morphology of the hybrid nanomaterial was studied via scanning electron microscopy (SEM) using a SU8020 Microscope from Hitachi (Tokio, Japan) at an operating voltage of 30 kV. In addition, transmission electron microscopy (TEM) using a JEM-1011 from Jeol Ltd. (Tokio, Japan) and high resolution TEM (Jeol 2100, 200 keV) equipped with an energy-dispersive X-ray spectrometer (EDXS) for elemental analysis were also performed. The specimens for high resolution TEM investigation were ultrasonically dispersed in MeOH and a drop of dispersion was deposited onto a lacy carbon film supported by a copper grid. Moreover, a drop of the IrO_x_ suspension was deposited onto a copper grid and studied by TEM.

The chemical composition of the hybrid sample was studied via X-ray photoelectron spectroscopy (XPS) using a VERSAPROBE PHI 5000 from Physical Electronics Inc. (Chanhassen, MN, USA), equipped with a Monochromatic Al Kα X-ray. The energy resolution was 0.6 eV. For the compensation of built charge during the measurements, a dual beam charge neutralization composed of an electron gun (~1 eV) and an Argon ion gun (<10 eV) was used. All binding energies were calibrated to the Au 4f_7/2_ (84.0 eV).

### 2.3. Sensor Fabrication

A silicon wafer was oxidized in a tubular furnace during 6 h at 1100 °C under continuous flow of dry oxygen. This process results in the growth of a silicon dioxide layer (SiO_2_) on both sides of the wafer. The wafer was diced. In the polished side of a given die, MWCNTs (either pristine or decorated with iridium oxide nanoparticles) were deposited by an airbrush technique, using a shadow mask. On the other side of the die (non-polished), a platinum screen-printed alumina heater was glued employing a thermally conductive epoxy and subsequently bonded to a 20 × 30 mm printed circuit board (PCB). Finally, two wire contacts were made on the surface of the sensor using a silver epoxy paste (Ag component metallization, Heraeus). The sensor layout was designed and implemented in order to be placed in a Teflon airtight chamber, which was connected to a computer controlled gas mixture and delivery system that employed mass-flow controllers from Bronkhorst High-Tech B.V. (Ruurlo, The Netherlands) and electro-valves (see Figure S2 Supplementary Materials).

### 2.4. Gas Sensing Studies

Calibrated gas cylinders of the different gases/vapors tested were employed diluted in a balance of synthetic dry air (Air Premier Purity: 99.995%). Pure dry air was also used as carrier gas. To achieve the desired analyte concentrations, successive dilutions were done using synthetic dry air. The total flow was adjusted to 100 mL/min, stabilizing the sensors with synthetic dry air during 1 h between exposures (30 min) to the target gas concentration. Sensor response is defined as (ΔR/R_0_) expressed in percentage, where ΔR is the resistance change over the 30-min exposure time and R_0_ is the baseline resistance. The time needed for achieving a stable sensor resistance value after a sudden exposure to nitrogen dioxide or ammonia exceeds one hour. In addition, recovering the initial baseline in dry air, especially after being exposed to nitrogen dioxide, takes a few hours. Therefore, to speed up the characterization process, the exposure and recovery times were arbitrarily set to 30 min and one hour, respectively. This implies that sensor responses reported are pessimistically biased, since allowing for the full stabilization during response and recovery would result in higher resistance changes than those reported. The different species were tested at three different operating temperatures (i.e., room temperature, 100 °C and 150 °C). Moreover, a controlled evaporator and mixer from Bronkhorst High-Tech B.V. (Ruurlo, The Netherlands) was used to humidify gas samples during the measurements, enabling the study of the relative humidity (R.H.) effect on sensor response. Resistance changes of the different gas sensitive films tested were monitored by an Agilent HP 34972A multimeter.

## 3. Results

At first, the morphology and composition of the hybrid gas sensitive nanomaterial was characterized employing Raman spectroscopy, SEM, TEM, and XPS.

### 3.1. Material Characterization Results

**Figure 1:**
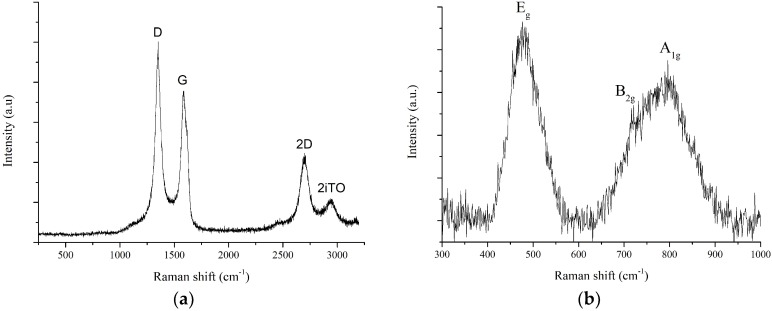
Typical Raman spectrum of the multi-wall carbon nanotubes (MWCNTs) used (**a**). Detailed Raman spectrum recorded in the 300–1000 cm^−1^ region corresponding to peaks attributed to the presence iridium oxide nanoparticles in IrO_x_-MWCNT samples (**b**).

Figure 1a shows the Raman spectrum of the hybrid nanomaterial with the well-known bands at 1350 cm^−1^ (D), 1580 cm^−1^ (G), 2680 cm^−1^ (2D), and 2950 cm^−1^ (2iTO). D and 2D band are related to the presence of defects such as disorder in the sp^2^ carbon nanostructure, amorphous carbon or carbonaceous impurities, meanwhile G band represents the in-plane vibrations of sp^2^ carbon bonds [20]. Taking in consideration the ratio between the intensities of D and G bands (D/G ratio), it was confirmed that the MWCNTs employed here are not highly crystalline. This is due to the presence of defects and oxygenated functional groups attached to the sidewalls of nanotubes resulting from the oxygen plasma treatment. However, the presence of these oxygenated defects plays an essential role in the anchoring process of IrO_x_ nanoparticles. Besides, the presence of oxygenated species in the surface of MWCNTs enhance their reactivity [16]. In other words, the presence of functional groups (i.e., COOH) contributes to increasing the sensitivity to gas molecules. In addition, Raman measurements revealed the decoration of MWCNTs with IrO_x_ (see Figure 1b), because the presence of IrO_x_ active modes (E_g_, B_2g_, and A_1g_) [21] could be detected.

**Figure 2:**
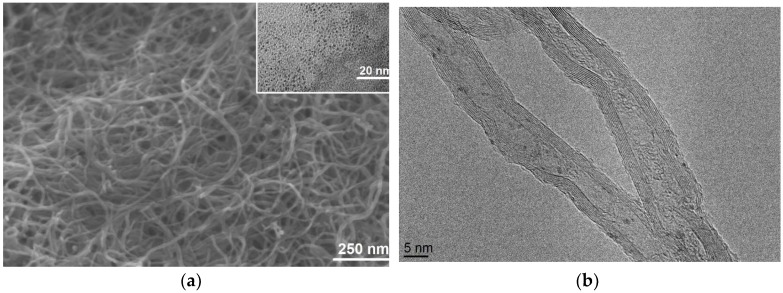
(**a**) SEM image showing the morphology of the IrO_x_-MWCNT sample. IrO_x_ nanoparticles (bright spots) can be observed at the MWCNT surface. The inset shows a TEM image of as synthesized iridium oxide nanoparticles (dark spots). (**b**) HR-TEM image showing MWCNT with IrO_x_ nanoparticles (dark spots).

SEM analysis shows that the hybrid nanomaterial consists of mats of disordered MWCNTs (Figure 2a). Some white spots appearing in the SEM micrograph (due to charge accumulation) can be attributed to the presence of semiconductor IrO_x_ nanoparticles sitting on the rather conductive MWCNTs. The synthesis method produced small nanoparticles, the size of which was 1 ± 0.3 nm (see Figure 2a inset). IrO_x_ nanoparticles appear as dark spots in TEM micrographs. The presence of IrO_x_ nanoparticles was further confirmed by HR-TEM and EDXS analysis (see Figure 2b and Figure S3 Supplementary Materials). The HR-TEM image also shows that the structure of the MWCNTs is preserved after the plasma and IrO_x_ impregnation treatments.

**Figure 3:**
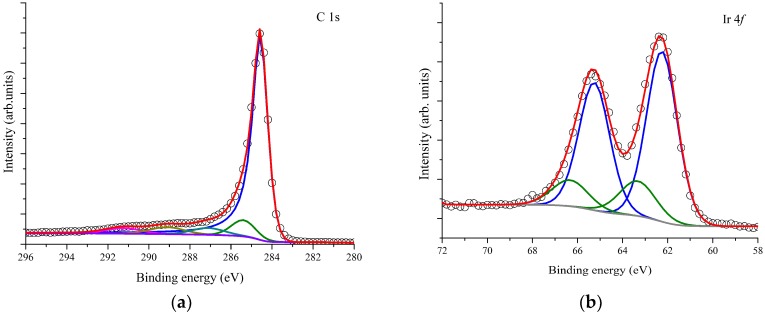
Deconvolution of the C 1s core level peak for bare MWCNTs (**a**). Deconvolution of the Ir 4f core level peak for iridium oxide nanoparticles (**b**).

The results of the XPS analysis are presented in Figure 3. The C1s spectrum is reproduced by five components centered at binding energy 284.4 eV, 285.5 eV, 287.2 eV, 288.9 eV, and 291.4 eV (Figure 3a). The components at 284.4 eV and 291.0 eV are characteristic of sp^2^ carbon systems, the first can be associated to photoelectrons emitted from carbon atoms in the carbon nanotube ‘graphite-like’ walls, while the second reflects the electron energy loss peak due to the collective excitation of π electrons, the so-called π plasmon [22,23]. The other three components are associated to photoelectrons emitted from carbon atoms at sp^3^ bonds, oxygen-containing groups such as C–O and in carboxylic groups, respectively [24].

Figure 3b shows a typical Ir 4f XPS spectrum recorded on the hybrid nanomaterial and its fitting result. Two doublets of Gaussian-Lorentzian convolution were used to reproduce the experimental data. The spin-orbit doublet binding energy splitting in each doublet was 2.9 eV and the intensity ratio 7:5. The first doublet with components at 62.3 eV (4f_7/2_) and 65.3 eV (4f_5/2_) testifies for the presence of Ir (III) while the second doublet with components centered at 63.4 eV and 66.4 eV the presence of Ir(IV) [25,26]. The fact that Ir presents two oxidation states is favourable for gas sensing, as will be discussed later.

### 3.2. Gas Sensing Results

The ability of the hybrid nanomaterial developed for detecting different gases was evaluated, showing significant results in the detection of NH_3_ and NO_2_ at ppm and ppb levels, respectively. Sensors employing decorated MWCNTs with IrO_x_ nanoparticles showed enhanced sensitivity, stability, and reproducibility than those employing bare carbon nanotubes.

**Figure 4:**
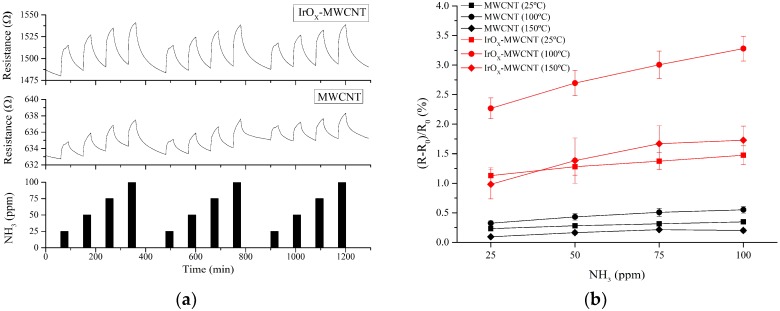
Typical resistance response obtained when detecting NH_3_ at a sensor operating temperature of 100 °C (**a**). Calibration curves obtained for NH_3_ at different operating temperatures (**b**).

NH_3_ detection was performed by analyzing repeated response and recovery cycles to four concentrations (25, 50, 75, and 100 ppm, successively), showing an important increase in response (six-fold) for IrO_x_ loaded carbon nanotubes, compared to bare carbon nanotube sensors. Moreover, apart from the higher response, IrO_x_-MWCNT presented a better reproducibility and higher stability (see Figure 4a). In addition, the responses were analyzed at three different sensor operating temperatures and IrO_x_-decorated MWCNT sensors showed higher ammonia responses than bare MWCNT sensors for any of the operating temperatures studied. The best working conditions were found to be 100 °C, considering the higher intensity of response and sensitivity (slope) achieved at this operating temperature (see Figure 4b). Error bars are standard deviations of responses.

**Figure 5:**
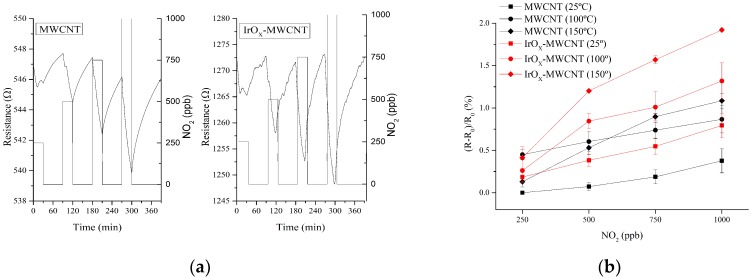
Typical response and recovery of the resistance signals when the sensors are exposed to different concentrations of NO_2_ (**a**). Calibration curves obtained for NO_2_ for bare carbon nanotubes and IrO_X_-MWCNTs at different operating temperatures under dry conditions. Error bars are standard deviations of responses (**b**).

The process followed for measuring NO_2_ was similar to the one employed for NH_3_. However, in this case four concentrations (see Figure 5a) were analyzed (250, 500, 750, and 1000 ppb), again for three different operating temperatures. It can be observed that a better responsiveness towards NO_2_ (two-fold increase) was obtained for MWCNTs loaded with IrO_x_ nanoparticles. Taking in consideration the responses obtained under different working conditions, the optimal operating temperature for detecting NO_2_ was established at 150 °C (see Figure 5b).

**Figure 6:**
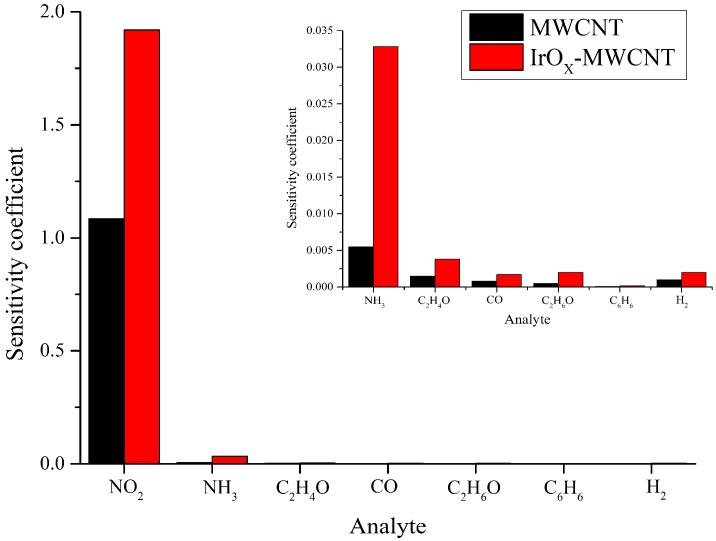
Sensitivity coefficient expressed as response/concentration for the different species tested (the inset is an enlargement showing the coefficients for those gaseous species with lower sensitivity coefficients). The sensitivity coefficients were obtained employing the highest response registered at specific concentrations, which were 1 ppm for NO_2_; 20 ppm for C_2_H_6_O and C_6_H_6_; 100 ppm for CO, C_2_H_4_O and NH_3_; and 1000 ppm for H_2_.

In addition, cross-sensitivity was evaluated measuring other gases. Some aromatic volatile organic compounds (VOCs), such as benzene and toluene were measured at low concentrations and at different operating temperatures without achieving high sensor responses during their exposure to these compounds. Exposure to ethanol (C_2_H_6_O) vapors did not resulted in significant response either. High concentration (100 ppm) of carbon monoxide (CO) was also tested, yet unsuccessfully. Besides, hydrogen was measured until high concentrations (e.g., 1000 ppm) resulting in extremely low response. A comparison of the responses obtained for the different gases tested can be observed in Figure 6. This figure reports a sensitivity coefficient that is defined as the ratio between the response measured and concentration tested for any given species. It can be derived that IrO_x_-MWCNTs are suitable for detecting NO_2_ and NH_3_ with small cross-sensitivity from other species.

Acetaldehyde (C_2_H_4_O) was also measured, obtaining a non-conclusive response for bare carbon nanotubes. However, decorated MWCNTs with iridium nanoparticles show a fast and saturated response when C_2_H_4_O is applied at 100 °C (Figure S4 Supplementary Materials), even at room temperature. However, an important drift can be observed together with a progressive de-sensitization effect over time, probably due to an irreversible adsorption by the repeated exposure of the gas-sensitive film towards C_2_H_4_O at low temperatures.

**Figure 7:**
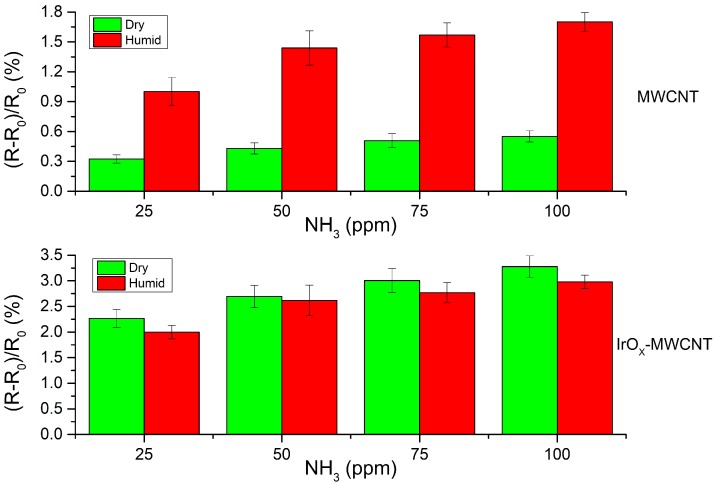
Comparison of the response towards ammonia for bare and IrO_x_-decorated MWCNTs under dry and humid (50% R.H.) conditions. Sensors were operated at 100 °C. For bare carbon nanotubes, the response towards ammonia shows a four-fold increase when under humid conditions. IrO_x_-MWCNTs present a slightly lower response to ammonia in humid conditions than in dry air.

The humidity effect on gas sensing performance was also analyzed. When the humidity background changes from dry to 50% R.H., the baseline resistance of IrO_x_-MWCNTs increases by 3.2% on average. Once this new baseline under humid conditions was reached and stable, which took about 5 min, ammonia and nitrogen dioxide measurements under humid conditions were performed. Bare carbon nanotubes show an important increase in the response towards nitrogen dioxide and ammonia when humidity is present, probably because of transfer of electronic charge from adsorbed water molecules towards carbon nanotubes depletes carbon nanotubes from holes in p-type carbon nanotube mats [27]. In contrast, during their exposure to NH_3_, the response of IrO_x_-decorated MWCNTs remains virtually unaffected by the presence of ambient moisture (see Figure 7). Even that a slight decrease in the response towards NH_3_ can be observed in Figure 7, this change falls within the range of the measurement uncertainty.

**Figure 8:**
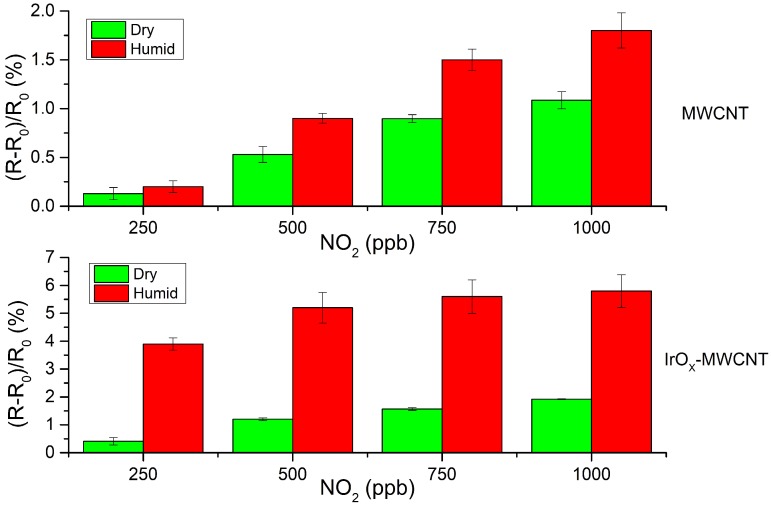
Comparison of the responses towards nitrogen dioxide of bare and IrO_x_-decorated MWCNTs under dry and humid (50% of relative humidity) conditions. Sensors were operated at 150 °C. While for bare carbon nanotubes, the response under humid conditions shows an almost two-fold increase (in comparison to dry conditions), this increase in response is even higher for IrO_x_-MWCNTs (nearly four-fold).

Similarly, the response to NO_2_ was evaluated in the presence of ambient moisture (50% R.H.) for a sensor working temperature of 150 °C. Figure 8 shows a high increase in the response towards NO_2_ of IrO_x_-MWCNTs when under humid conditions. Figure S5 (Supplementary Materials) shows a typical dynamic response of a sensor for nitrogen dioxide under humid conditions. The possible gas sensing mechanism to explain this effect will be detailed later on, in the discussion section.

The long term stability of the response towards nitrogen dioxide was studied as well. For this purpose, repeated nitrogen dioxide measurements were conducted at 1 ppm under dry conditions for both IrO_x_ loaded and bare MWCNT sensors over a 6-month period. It was found that the response of IrO_x_-MWCNTs was remarkably stable (variation was below 5%). Figure S6 (Supplementary Materials) shows the details.

It is important to notice that IrO_x_-MWCNT sensors are able to measure ppm and ppb levels of NH_3_ and NO_2_, respectively, with an excellent signal-to-noise ratio. For that reason, it is interesting to estimate the limit of detection (LOD) and limit of quantification (LOQ). In the case of ammonia, it was considered applying a linear regression to the calibration curve, following the method described by Shrivastava and co-workers [28]:

LOD = 3*S_a_*/*b* (2)

LOQ = 10*S_a_*/*b* (3)

where *S_a_* was estimated by the standard deviation of y-intercepts and *b* is the slope of the regression line obtained from Figure 4b. The theoretical LOD and LOQ for IrO_x_-MWCNTs were estimated at hundreds of ppb for NH_3_ detection. These results should be confirmed with measurements at ppb range, however, these levels are much lower than the exposure limit [29] established by the Occupational Safety and Health Administration (OSHA), which is 50 ppm as an 8-h time-weighted average (TWA).

**Figure 9:**
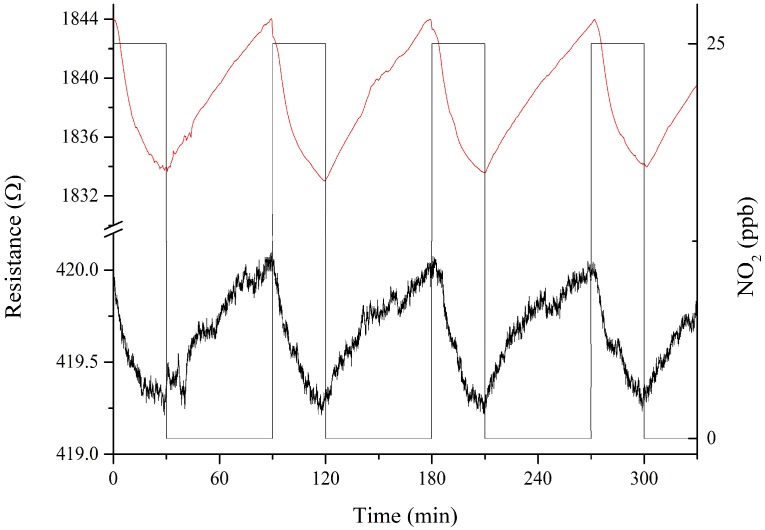
Example of the response for bare carbon nanotubes (black line) and IrO_x_-MWCNTs (red line) to repeated pulses of nitrogen dioxide at 25 ppb. Sensors were operated at 150 °C. A baseline correction was implemented to suppress baseline drift.

Carbon nanomaterials present high potential to detect very low concentrations of nitrogen dioxide. For that reason, 25 ppb of NO_2_ were measured (see Figure 9) to check the ability of the sensors to reproducibly detect such a low concentration level. This is the lowest NO_2_ concentration that our measurement system is able to generate.

To estimate the LOD and LOQ for nitrogen dioxide, a signal-to-noise method was employed [30]. The background noise level was calculated using 50 points during a stabilization step under a flow of dry air. In addition, for each sensor, the response signal for 25 ppb of nitrogen dioxide was computed as the averaged response for 10 nitrogen dioxide pulses. This is illustrated for four pulses in Figure 9. Then, the sensitivity was estimated using the slope of the calibration curve shown in Figure 5b (for the two lowest concentrations measured, i.e., 250 and 500 ppb) obtaining a sensitivity of 0.0032% ppb^−1^ and 0.0015% ppb^−1^ for iridium oxide decorated and bare MWCNTs, respectively. Assuming a signal-to-noise ratio of 3 for LOD, and of 10 for LOQ, the estimated levels are summarized in Table 1. The lower values obtained with IrO_x_ loaded MWCNTs are due to the higher response and signal-to-noise ratio for this hybrid nanomaterial than for bare MWCNTs.

Maximum permitted exposure limits to NO_2_ are under continuous revision, but nowadays these are established at 200 ppb and 100 ppb (1-h exposure) by the European Union (EU) [31] and the US [32], respectively. Besides, the annual limit mean for primary and secondary exposure is 40 ppb for the EU [31] and 53 ppb for US [32]. Our sensors show clearly the possibility of detecting 25 ppb of nitrogen dioxide and potential for detecting this toxic species even at lower levels. Nevertheless, real exposures to these concentrations of NO_2_ should be studied in order to confirm the theoretical LOD and LOQ obtained.

## 4. Discussion

Despite the fact that carbon nanotube mats can work at room temperature for gas sensing [33], the presence of metal oxide nanoparticles, which may show catalytic properties above room temperature, has encouraged us to explore the performance of the hybrid nanomaterials at moderate operating temperatures (up to 150 °C). This should help us better apprehend the effect of IrO_x_ nanoparticles decorating MWCNTs on gas sensing properties [34]. From the gas sensing tests, it was derived that the presence of IrO_x_ NPs decorating the outer wall of MWCNTs was advantageous for detecting ammonia and nitrogen dioxide. Additionally, ammonia was better detected at an operating temperature of 100 °C, while 150 °C was better for detecting nitrogen dioxide. While MWCNTs offer oxygenated-defect sites and carboxylic acid functional groups to interact with these species, IrO_x_ nanoparticles present a high content of oxygen species on their surface, improving the sensitivity to some gases.

Ammonia is a reducing agent, acting as electron donor. Then, when p-type carbon nanotube mats interact with NH_3_, an increase in the film resistance is observed because electronic charge is transferred from adsorbed ammonia molecules towards CNTs [35]. The presence of oxygenated defects on the surface of CNTs favours their interaction with ammonia [36]. In contrast, nitrogen dioxide is a strong oxidizing agent with electrophilic properties, acting as electron acceptor. As a consequence, when nitrogen dioxide is adsorbed on carbon nanotubes, electronic charge is transferred from CNTs towards the adsorbed species and the electrical resistance of the mat decreases. As already discussed for ammonia, oxygenated defects can act as adsorption sites [37]. These mechanisms are detailed below:

NH_3 (gas)_ → NH_3_^+^_(ads)_ + e^−^ (4)

NO_2 (gas)_ + e^−^ → NO_2_^−^_(ads)_ (5)

At moderate temperatures, the adsorption barrier is further lowered by the presence of oxygen via adsorbed molecular oxygen from the environment and the oxygenated species and defects present on the carbon nanotube sidewalls [20]. These oxygenated species on MWCNTs can be attributed to the presence of functional groups resulting from the plasma treatment and adsorbed oxygen from the sensor environment during the experiments [38].

Moreover, independently of the operating temperature, decorated carbon nanotubes always show a higher response to ammonia or nitrogen dioxide than bare MWCNTs. The presence of IrO_x_ nanoparticles improves the response and sensitivity offered by carbon nanotubes. The interactions described between gases and oxygenated species for pristine carbon nanotubes can be applied to IrO_x_ nanoparticles as well. Nanoparticles of oxygen defective iridium oxide (as revealed by XPS) facilitate the interaction between gas molecules and adsorbed oxygen species (O_2_^−^) [36], which results in the transfer of electronic charge between adsorbed molecules and the NP-MWCNT system.

Nitrogen dioxide and ammonia can react with the oxygen species adsorbed at metal oxide nanoparticles, following the reactions proposed by Rahmani and co-workers [39]:

4NH_3 (gas)_ + 3O_2_^−^ → 2N_2 (gas)_ + 6H_2_O + 3e^−^ (6)

NO_2 (gas)_ + O_2_^−^ + 2e^−^ → NO_2_^−^_(ads)_ + 2O^−^_(ads)_ (7)

**Figure 10:**
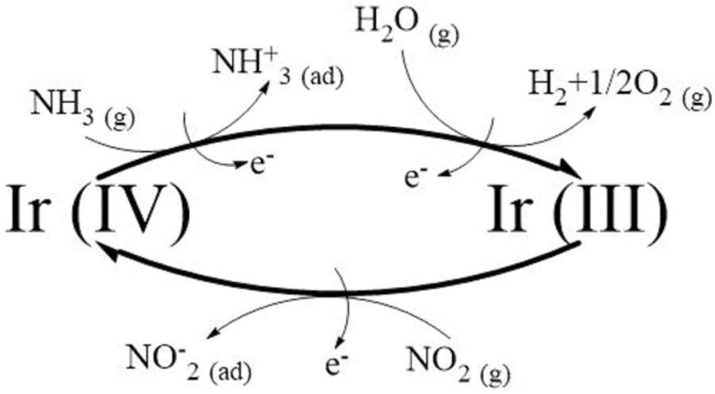
Gas sensing mechanism for iridium oxide nanoparticles decorating carbon nanotubes.

The release (capture) of electrons upon adsorption of ammonia (nitrogen dioxide) results in the increase (decrease) of the electrical resistance of the IrO_x_-MWCNT mats. In addition, iridium oxide has been reported as a catalytic material, and the XPS analysis conducted on iridium oxide decorated MWCNT samples (see Figure 3) has shown that two oxidation states coexist for Ir. The presence of both Ir (IV) and Ir (III) was determined, which means that NPs contain IrO_2_ and Ir_2_O_3_. The coexistence of these two iridium oxides could explain the high response towards ammonia and nitrogen dioxide obtained for IrO_x_-decorated MWCNT samples in comparison to bare MWCNT samples. The gas sensing mechanism that we propose, derived from the occurrence of different oxidation states for iridium is detailed in Figure 10.

Probably iridium oxide nanoparticles present a redox interaction with the analytes, which means that Ir (IV) is reduced by NH_3_ going to Ir (III) state. However, NO_2_ can oxidize Ir (III) to Ir (IV). These interactions could explain the higher sensitivity to these gases observed for IrO_x_-MWCNTs. This is based on the presence of Ir (IV) and Ir (III) in the hybrid sensing material at the same time but in different ratios, depending on the gas tested.

We can consider now the presence of ambient moisture in the sensing mechanism. The high sensitivity of bare nanotubes to ambient moisture is well-known. In fact, carbon nanomaterials such as graphene and carbon nanotubes have been extensively reported as humidity sensors [40,41,42]. Here the enhancement in the response towards nitrogen dioxide or ammonia observed for bare MWCNT sensors under humid conditions can be attributed to a water mediated adsorption of gas molecules in semiconductor chemoresistors [43]. However, in IrO_x_-decorated carbon nanotubes the interactions with humidity are more complex. First, IrO_x_-MWCNTs show a similar response to ammonia under dry or humid conditions, even with ambient moisture the response is slightly lower. Probably the reason for this behaviour is related to the reducing properties of water (see Figure 10):

H_2_O _(gas)_ → 2H^+^_(gas)_ + 1/2O_2 (gas)_ + 2e^−^ (8)

At 100 °C, which was found optimal for detecting ammonia, IrO_x_ NPs are able to create a water splitting effect, reducing Ir (IV) to Ir (III). In consequence, reducing molecules such as NH_3_ and H_2_O are taking part in a competitive reaction that favours the reduction of IrO_x_ towards Ir_2_O_3_, which would explain the similar response observed for ammonia under dry or humid conditions due to the limitation in the number of surface oxygen species to interact with. In contrast, IrO_x_-MWCNTs show a higher response to NO_2_ in a humid environment than in dry air. Figure 10 can explain this behaviour because during any recovery phase under humid air, the occurrence of Ir (III) is favoured, increasing the Ir (III)/Ir (IV) ratio. This higher ratio explains the higher response recorded for a new NO_2_ exposure event.

Table 2 and Table 3 summarize the performance achieved with previously reported metal or metal oxide decorated carbon nanotube materials in the detection of NO_2_ and NH_3_, respectively. In addition, these tables also help putting in context the results achieved using IrO_x_-decorated carbon nanotubes. As described above, the operational sensitivity reported in these tables was estimated using the slope of the calibration curves for the lowest concentrations measured.

## 5. Conclusions

A p-type chemoresistive sensor based on IrO_x_ nanoparticles decorating MWCNTs was devised to successfully detect harmful gases like NO_2_ and NH_3_ at different working temperatures. These loaded carbon nanotubes show enhanced gas sensing properties, such as better reproducibility, higher sensitivity, stability, and lower noise levels in comparison to their bare MWCNT counterparts. In addition, the effect of relative humidity on sensor response was studied, and a detailed gas sensing mechanism was proposed to understand the influence of ambient moisture in the presence of a catalytic nanomaterial like iridium oxide nanoparticles. Finally, low level of cross-sensitivity was observed for a range of different gases and vapors with interfering potential. In consequence, IrO_x_-MWCNT nanomaterial enables quite a selective detection of nitrogen dioxide or ammonia against other hazardous gases, with low detection limits, making it a potential nanomaterial to be employed in real applications.

## Figures and Tables

**Table 1:** Estimated limits of detection and quantification for NO_2_. These theoretical levels should be confirmed with experimental measurements at this level of concentration.

	CNT	IrOx-MWCNT
LOD	17.8 ppb	1 ppb
LOQ	59.1 ppb	3.2 ppb

**Table 2:** Nitrogen dioxide sensitivities reported as 10^−3^% ppb^−1^ for different metal or metal oxide nanoparticles decorating carbon nanotubes. TW = This Work.

CNT Decoration	Sensitivity	Reference
IrO_x_	3.2	TW
Au	8	[44]
Rh	5	[37]
Pt	0.094	[45]
Pd	0.069	[45]
SnO_2_	4.8	[46]
ZnO_2_	0.25	[47]

**Table 3:** Ammonia sensitivities reported as 10^−2^% ppm^−1^ for different metal nanoparticles decorating carbon nanotubes. TW = This Work.

CNT Decoration	Sensitivity	Reference
IrO_x_	1.71	TW
Co	0.36	[48]
Au	0.41	[49]
Pd	1.11	[50]
Pt	2.80	[51]
Ag	6.84	[51]

## Supplementary Materials

The following are available online at http://www.mdpi.com/1424-8220/19/1/113/s1. Figure S1: summary of IrO_x_ NPs synthesis, Figure S2: Gas testing chamber and design of sensor used, Figure S3: TEM-EDXS spectrum of the IrO_x_-MWCNT sample, Figure S4: Acetaldehyde detection, Figure S5: Response to NO_2_ in humid conditions for IrO_x_-MWCNTs, Table S1: Average responses and associated standard deviations, Figure S6: Response stability test.
