# Supplementary material for: Gas Sensing with Iridium Oxide Nanoparticle Decorated Carbon Nanotubes
Source: Sensors (Basel). 2018 Dec 31;19(1):113. doi: 10.3390/s19010113 (PMC6339137; doi:10.3390/s19010113)
Supplement: Supplementary file 1 [file sensors-19-00113-s001.pdf]

SUPPLEMENTARY MATERIAL for

Gas Sensing with Iridium Oxide Nanoparticle Decorated Carbon Nanotubes

Juan Casanova-Cháfer [1], Eric Navarrete [1], Xavier Noirfalise [2], Polona Umek [3], Carla Bittencourt [4] and Eduard Llobet [1,*]

[1] MINOS-EMaS, Universitat Rovira i Virgili, 43007 Tarragona, Spain; juan.casanova@urv.cat (J.C.-C.); eric.navarrete@urv.cat (E.N.)
[2] Materia Nova, 7000 Mons, Belgium; xavier.noirfalise@umons.ac.be
[3] Jožef Stefan Institute, 10000 Ljubljana, Slovenia; polona.umek@ijs.si
[4] ChIPS, University of Mons, 7000 Mons, Belgium; carla.bittencourt@umons.ac.be
* Correspondence: eduard.llobet@urv.cat; Tel.: +34-997-558-502

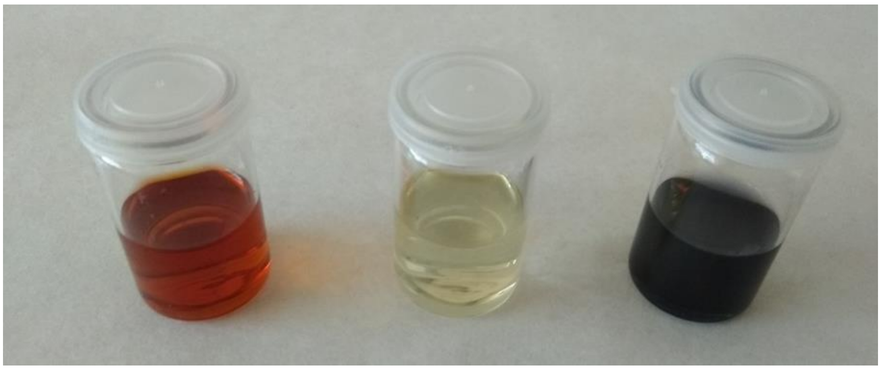

Figure S1. Steps to synthetize iridium nanoparticles ligand free. From left to right, red-brown solution from K_2IrCl_6 diluted in distilled water; yellow color due to the break of Ir-Cl bonds, creating the $[Ir(OH)_6]^{-2}$ complex; and finally, deep blue obtained after the acid condensation, obtaining IrO_x nanoparticles ligand free.

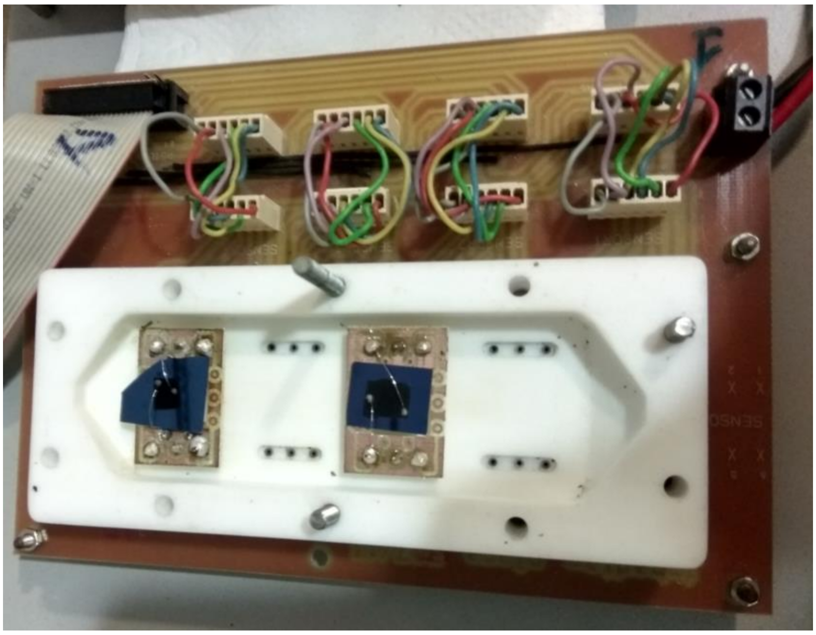

Figure S2. Airtight Teflon chamber with IrO_x-MWCNTs deposited on a silicon wafer and connected to a PCB.

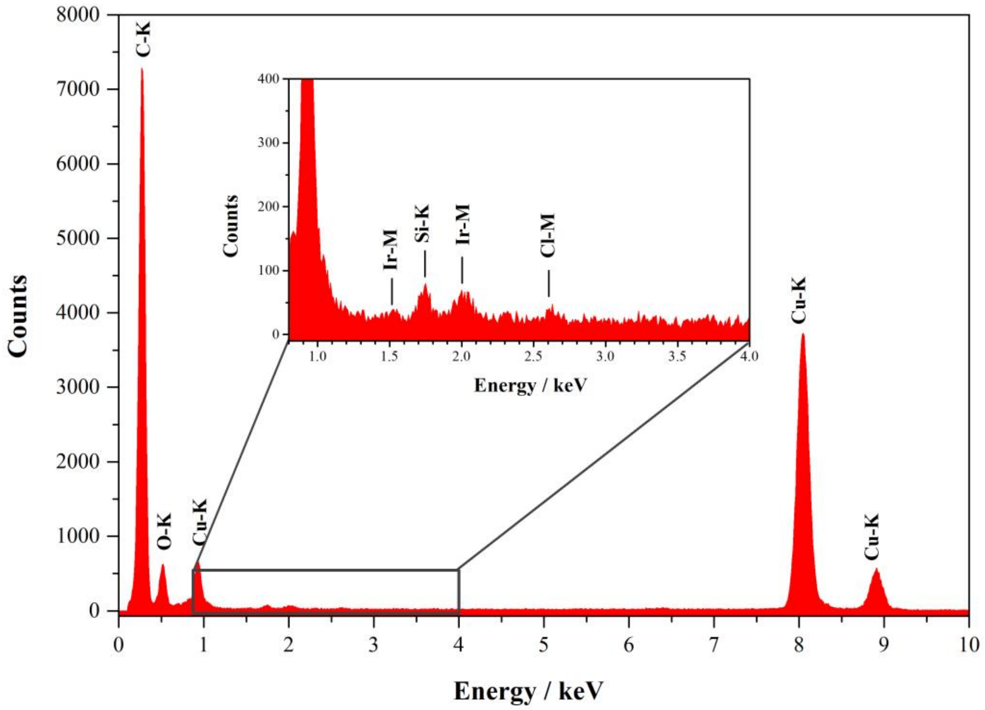

Figure S3. TEM-EDXS spectrum of the IrOx-MWCNT sample. Spectrum was taken over the area shown in the TEM image presented in Figure 2 (bottom panel). Beside Ir-M peak in the spectrum are also present Si-K and Cl-M peaks. Chlorine comes from the IrOx precursor (Equation (1)) while silicon from the glass. Signals for copper and carbon arise from the TEM grid.

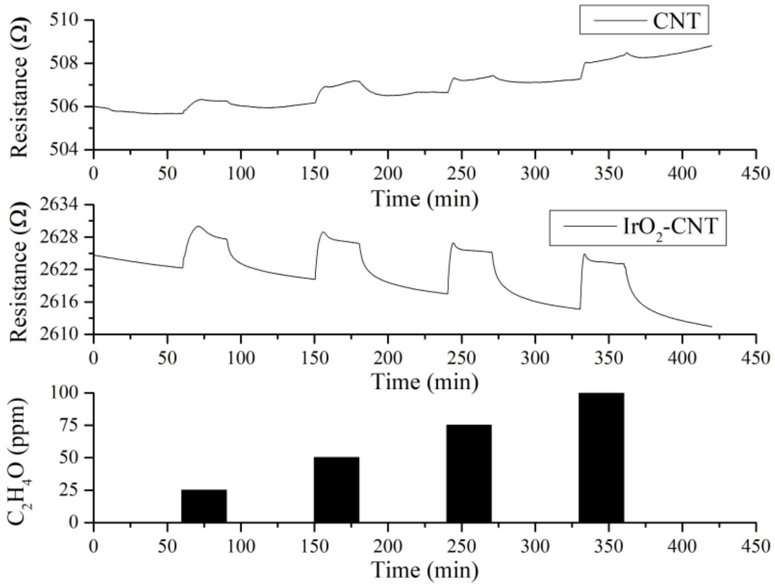

Figure S4. Acetaldehyde detection at 100 °C.

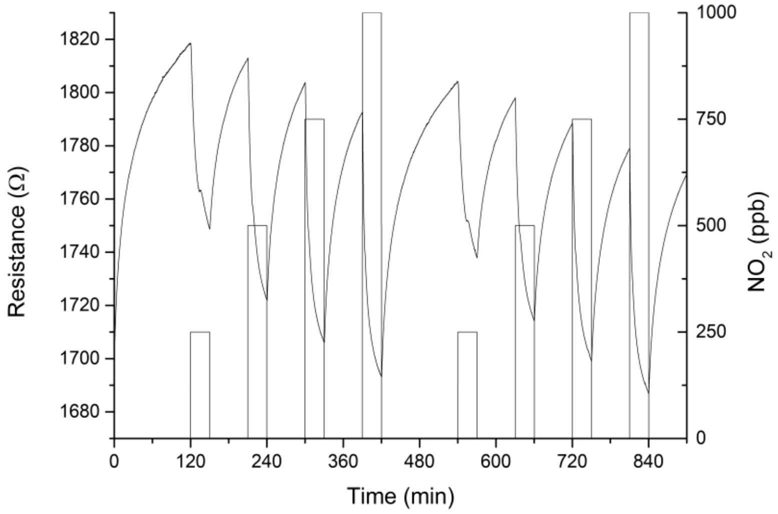

Figure S5. Example of response to NO_2 for IrO_x-MWCNT under 50% of relative humidity and 150 °C.

Table S1. Average sensor responses and their associated standard deviations for nitrogen dioxide and ammonia employing the optimum working temperatures.

NO_2 (ppb)	MWCNT	IrO_x-MWCNT
250	0.13 ± 0.06	0.41 ± 0.11
500	0.53 ± 0.08	1.20 ± 0.05
750	0.90 ± 0.04	1.57 ± 0.05
1000	1.09 ± 0.09	1.92 ± 0.012

NH_3 (ppm)	MWCNT	IrO_x-MWCNT
25	0.32 ± 0.04	2.27 ± 0.18
50	0.43 ± 0.06	2.70 ± 0.21
75	0.51 ± 0.07	3.00 ± 0.23
100	0.55 ± 0.06	3.28 ± 0.21

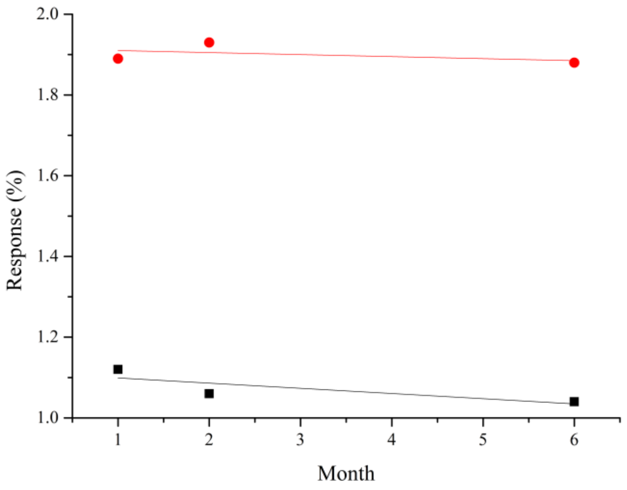

Figure S6. Long term stability test for the detection of nitrogen dioxide (1 ppm) under dry conditions. Red is for an IrOx-MWCNT sensor and black for a bare MWCNT sensor. After six months of use, the IrOx-MWCNT sensor shows a remarkable response stability.
